# Treatment with 24 h–delayed normo- and hyperbaric oxygenation in severe sepsis induced by cecal ligation and puncture in rats

**DOI:** 10.1186/s12950-017-0173-4

**Published:** 2017-11-25

**Authors:** Nina Falcon Bærnthsen, Marco Bo Hansen, Anna Mygind Wahl, Ulf Simonsen, Ole Hyldegaard

**Affiliations:** 1 Department of Anesthesia, Center of Head and Orthopedics, Rigshospitalet, University of Copenhagen, Blegdamsvej 9, DK-2100 Copenhagen, Denmark; 2 Hyperbaric Unit, Department of Anesthesia, Center of Head and Orthopedics, Rigshospitalet, University of Copenhagen, Blegdamsvej 9, DK-2100 Copenhagen, Denmark; 3 Department of Biomedicine, Pulmonary and Cardiovascular Pharmacology, Aarhus University, Nordre Ringgade 1, DK-8000 Aarhus, Denmark

**Keywords:** Sepsis, Mortality, CLP, Cytokines, Inflammation, HBO_2_

## Abstract

**Background:**

Septic shock remains a leading cause of death worldwide. Hyperbaric oxygen treatment (HBO_2_) has been shown to alter the inflammatory response during sepsis and to reduce mortality. A therapeutic window of HBO_2_ treatment has been demonstrated experimentally, but optimal timing remains uncertain. We investigated the effects of 24 h delayed normobaric oxygen (NBO_2_) and HBO_2_ treatment on the endogenous production of the inflammatory markers interleukin (IL)-6, tumor necrosis factor (TNF)-α and IL-10, and on mortality in rats with cecal ligation and puncture (CLP) induced sepsis.

**Method:**

Fifty-five male Sprague-Dawley rats underwent CLP and were randomized to the following groups: 1) HBO_2_ 2.5 bar absolute pressure (p_abs_); 2) NBO_2_ 1.0 bar p_abs_; 3) Control (no-treatment), and they were individually monitored for 72 h with intermittent blood sampling.

**Results:**

IL-6, TNF-α, and IL-10 were increased 24 h after the procedure, and IL-6 was significantly higher in non-survivors than in survivors. The level of IL-10 was significantly higher at hour 48 in the HBO_2_ group compared to control (*p* = 0.01), but this was not the case at other time points. No other significant differences in cytokine levels were found for any group comparisons. Delayed NBO_2_ and HBO_2_ treatment failed to change the mortality in the animals.

**Conclusion:**

High levels of IL-6 in non-surviving animals with sepsis suggest that IL-6 is a potential biomarker. We found a significantly higher concentration of IL-10 in the HBO_2_ group at hour 48 vs. control animals. However, 24 h–delayed treatment with HBO_2_ did not change the levels of pro-inflammatory cytokines and survival, suggesting that earlier intervention may be required to obtain an anti-inflammatory effect.

## Background

Sepsis is a common and critical illness that accounts for 750.000 deaths annually in the United States despite increased focus and improved treatment [1–4]. The excessive release of cytokines causes a hyper inflammatory state and the imbalance of the pro-inflammatory and anti-inflammatory responses is thought to contribute to the high mortality rates in sepsis [5]. In particular, high concentrations of tumor necrosis factor (TNF)-α and interleukin (IL)-6 have been associated with multi organ failure and death at Day 28 [6]. Expression of IL-10 has been suggested to protect against mortality [7, 8]. However, others have shown that mortality is highest when both anti-inflammatory and pro-inflammatory cytokine levels are elevated [5, 9].

Hyperbaric oxygen treatment (HBO_2_) is currently the primary treatment for patients with carbon monoxide poisoning, decompression sickness, and an adjunctive treatment for problematic wound healing including necrotizing soft tissue infections [10, 11]. Sepsis is associated with an increased formation of reactive oxygen species (ROS) involved in tissue damage. Even though hyperoxia might increase ROS production, hyperbaric hyperoxia triggered repair mechanisms such as increasing the antioxidative capacity and improving microcirculation seems of importance, and therefore HBO_2_ has been suggested as a treatment for sepsis [12, 13]. Data from animal studies of sepsis suggest that HBO_2_ reduces mortality by altering the inflammatory response with an up-regulated anti-inflammatory response and down-regulated pro-inflammatory response [14, 15]. Previous reports have established that early treatment with hyperbaric oxygen has a beneficial effect on anti-inflammatory parameters and on mortality, but at present it is unclear whether delayed intervention with HBO_2_ has effect on the manifest stage of septic shock [7, 16, 17]. Moreover, cytokine concentrations are often only measured at one time point in each animal e.g. prior to euthanization, thus eliminating the chance of investigating the dynamic changes in the individual animal over time [7, 18, 19].

In the present study we aimed to investigate whether sampling and monitoring cytokine levels at several time points during 72 h relate to mortality and whether delayed treatment with normo- or hyperbaric oxygenation (i.e. NBO_2_ or HBO_2_) has a modulating effect on the cytokine response in manifest sepsis. CLP-induced sepsis in rats is considered the gold standard for the induction of inflammatory responses because the method causes a septic response similar to that of humans [20, 21]. Therefore, we combined CLP-induced sepsis with intermittent blood sampling monitoring the endogenous production of IL-6, TNF-α and IL-10 in rats over 72 h. We hypothesized that HBO_2_ treatment would result in a decreased pro-inflammatory response and an increased anti-inflammatory response thereby lowering mortality.

## Methods

### Ethics

The study was approved by the Danish Animal Experiment Inspectorate (authorization number 2012–12–2934-00504). The animals were assessed every 8th hour by the investigator (NFB) for clinical signs of sepsis. If an animal was too severely affected, it had to be euthanized according to Danish law. The severity of sepsis was evaluated using objective criteria according to clinical observations including the rats alertness [22]. Signs of severe suffering due to sepsis should be handled with euthanization of the rat, although this never happened. The study complied with the national guidelines for the care and use of animals in experimental research. The manuscript was prepared according to the ARRIVE (Animal Research: Reporting of In Vivo Experiments) guidelines.

### Animals

Male Sprague-Dawley rats aged 8–10 weeks with an average weight of 296 g were used in all experiments. The rats were maintained, single caged, in a controlled environment (room temperature 22–23 °C and 50% humidity). They were housed for 120 h before surgery to allow acclimatization to the environment and they were maintained under a 12 h light/12 h dark regime. Food and water were available ad libitum.

### Surgical procedures

All surgical procedures were conducted under aseptic conditions. Body temperature was controlled with a heating pad to maintain body temperature close to 37 °C degrees. Body temperature was measured with a probe inserted in the rectum. The rats were anesthetized with a single shot of intramuscular administered Zoletil 0.25 mL/100 g to keep the animals asleep and pain free during the entire surgical procedure. The animals were anesthetized a total of two times during the research period: Once prior to CLP and once before termination after 72 h to allow cardiac puncture. All incisions were closed using 4–0 PDS*II suture. Post-surgery fluid resuscitation was administered once with saline 20 mL/kg intraperitonally and analgesia was provided with buprenorphine 0.05 mg/kg every 8 h.

#### Femoral venous catheter

While anesthetized, using aseptic technique, a femoral venous catheter was placed for blood sampling, tunneled under the skin to the back of the neck and placed in a harness (Instech Solomon, order no. CIH95AB) to be easy accessible for sampling. The catheter was placed through a 5 mm incision in the right inguinal area. The femoral vein was found using blunt dissection and the tunnel was made by guiding forceps subcutaneously to the level of the inguinal area. A small incision was made 1/3 through the vein at an angle of 45 degrees. The catheter was placed in the vein and forwarded approximately 2–3 cm into the vein. Afterwards it was secured with 5–0 ethilon*II suture in the muscle layer of the leg. After insertion and blood sampling the catheter was flushed with 1 mL of saline also functioning as fluid resuscitation. A total of 3 mL was drawn during the 72 h observation period and before the rat was anesthetized for termination corresponding to approximately 15% of the total blood volume [22]. This is considered in accordance to guidelines and should not cause significant circulatory- or oxygenation problems since blood is sampled over several days [23].

#### Cecal ligation and puncture

The rat was shaved in the surgical area before the procedure and the skin sterilized with 0.5% chlorhexidine gluconate 83% ethanol. A 2 cm midline incision was made in the abdominal wall using scissors. The cecum was located and 30% of the cecum length was tightly ligated with a 4–0 silk suture, the ligation being below the ileocecal valve to ensure the continuity of the gastrointestinal tract and otherwise according the procedures described by Rittirsch et al. and Hubbard et al. [21, 24]. The cecum was then punctured once from side to side (double puncture) with a 16G needle. The cecum was gently squeezed until a small amount (droplet) of feces was visible through the punctures. The cecum was relocated into the abdominal cavity without spreading feces to the abdominal wall wound and the abdominal wall and skin was closed by sutures using 4–0 PDS*II suture. After the rat recovered from anesthesia, they had access to food and water ad libitum.

### Experimental design and protocol

We randomized 55 rats into the following three groups, all rats undergoing CLP:

1. 19 rats exposed to hyperbaric oxygen (HBO_2_) (100% oxygen; 2.5 bar absolute pressure (p_abs_)).
2. 18 rats exposed to normobaric oxygen (NBO_2_) (100% oxygen; 1.0 bar p_abs_).
3. 18 rats served as controls (no treatment).

**Fig. 1:**
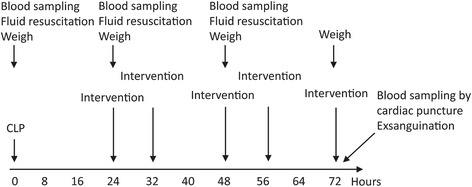
Experimental protocol showing interventions on a timeline. Intervention being either hyperbaric oxygen treatment, normobaric oxygen treatment or no intervention (control). CLP, cecal ligation and puncture. Blood = 1 ml of full blood withdrawn for analysis and substituted by 1 ml 0.9% NaCl

Rats were randomized (via Microsoft Excel) on a weekly basis amongst all valid combinations, the same group not following each other thereby assuring random group allocation. All normo- or hyperbaric oxygen treatments lasted for 90 min, the first treatment being administered with a delay of 24 h from the time of CLP. Subsequently, NBO_2_ or HBO_2_ were administered at 32, 48, 56 and 72 h after CLP, i.e. the last NBO_2_ or HBO_2_ treatment were followed by immediate termination by exsanguination (Fig. 1).

One mL of blood was aseptically collected from the femoral vein catheter immediately prior to CLP and subsequently at hour 24, 48 and 72 after CLP. After each sample was collected, 1 mL of saline was carefully administered over one minute to compensate for the blood collected. If the catheter was obstructed, this was recorded, blood was drawn via sublingual blood collection using a 23G syringe dripping the blood directly into the sample tube, here giving 1 mL of saline intramuscular At 72 h, the animals were anesthetized with Zoletil 0.25 mL/100 g and blood for culturing and cytokine measurements were drawn by cardiac puncture under sterile conditions. Immediately afterwards, all animals were terminated by exsanguination.

### Hyperbaric oxygen treatment

We administered 100% O_2_ for 90 min at 1.0 or 2.5 bar absolute pressure. The treatments were given in an Oxycom 250 Arc cylindrical acrylic hyperbaric chamber designed for animal research. The pressure inside the chamber was increased at the rate of 0.3 bar/min and decompressed at the rate of 0.3 bar/min. In the normobaric treatment the chamber was only ventilated with oxygen without increasing the pressure inside the chamber. The concentration of O_2_ was continuously measured with a DAMECA OM781 oxygen monitor. The hyperbaric chamber was ventilated with pure oxygen during the entire treatment to prevent CO_2_ accumulation and to maintain the exact pressure.

### Laboratory methods

#### Blood cultures

One-3 mL of blood was inoculated in Bactec™ Peds Plus culture vials for aerobic microorganisms. Subsequent culturing and identification of the bacterial isolates was performed according to standard microbiological methods by the Department of Clinical Microbiology, Sect. 9301, Copenhagen University Hospital, Rigshospitalet.

#### Luminex multiplex assay

Quantitative determination of IL-6, TNF-α and IL-10 concentrations in plasma was done using BioRad Bioplex System, Luminex MAP Technology (Copenhagen, Denmark) according to the manufacturer’s specifications. Analysis was performed at Aarhus University Hospital and at the Department of Biomedicine, Aarhus University. The plasma was obtained from whole blood centrifuged at 2400G/3500 rpm for 10 min. The plasma supernatant was pipetted and placed in 1 mL cryo tubes stored at minus 80 degrees Celsius for later analysis.

### Outcome measures

Our primary analysis focused on describing changes of the pro- and anti-inflammatory response during the development of CLP induced sepsis expressed as differences in IL-6, TNF-α and IL-10 concentrations. Furthermore, we assessed the impact of HBO_2_ and NBO_2_ treatments on plasma cytokine concentrations at 24 h, 48 h and 72 h during the phases of sepsis progression. In the secondary analysis, we analyzed differences in mortality over 72 h between the HBO_2_ and NBO_2_ group.

### Data analysis and statistics

**Fig. 2:**
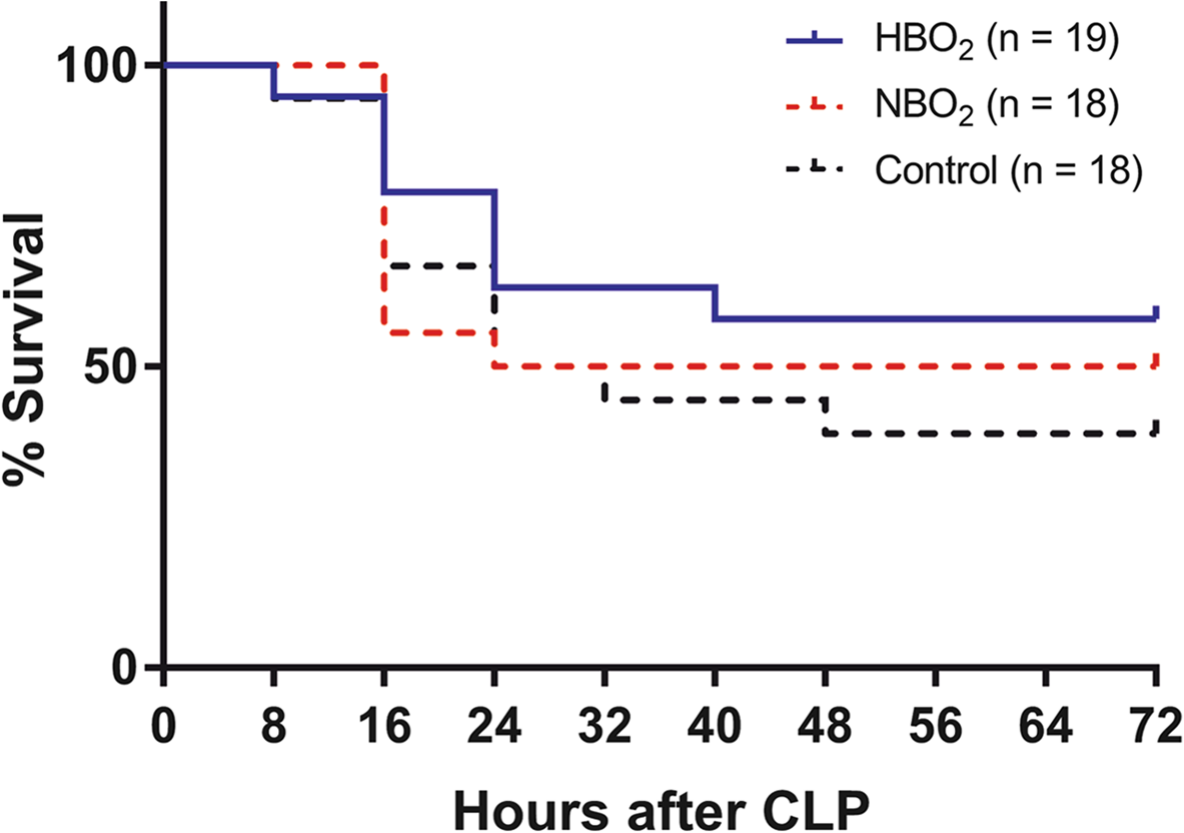
Kaplan-Meier curves showing the percentage survival in HBO_2_, NBO_2_ and control group during 72 h. HBO_2_, hyperbaric oxygen treatment; NBO_2_, normobaric oxygen treatment. HBO_2_ group compared to NBO_2_ group *p* = 0.59. HBO_2_ group compared to the control group *p* = 0.24. NBO_2_ group compared to the control group *p* = 0.59. Groups are compared using log-rank test

Tests for normality and homogeneity of variance were conducted using the Shapiro-Wilks test and the Kolmogorov-Smirnov test. Due to non-parametric distribution, continuous data are reported as medians (interquartile range). For categorical data, absolute numbers (proportions) are reported and group comparisons performed using χ^2^-test or Fisher’s exact test. Continuous data are reported with medians (interquartile range, IQR) and compared at specific time points using the Mann-Whitney *U* test. The mortality was calculated using the log-rank test and illustrated with Kaplan-Meier curves (Fig. 2). *P-*values <0.05 were considered statistically significant. Statistical analyses were performed using Statistical Package for the Social Sciences 22.0 software (SPSS Inc., Chicago, IL, USA) and GraphPad Prism 6.0 software (GraphPad Inc., La Jolla, CA, USA). Cytokine measurements with values lower than detection concentrations were set to 0.5 times the lowest value measured for statistical analysis.

## Results

In total, 58 rats underwent operation with CLP and insertion of femoral vein catheter. Three died as a result of operative complications, leaving 55 rats for inclusion. Baseline characteristics of all experimental groups are shown in Table 1. Except for weight, there were no differences among groups in baseline characteristics. In spite of group randomization procedures, the weight of the rats was significantly different among the groups with rats in the HBO_2_ (median weight 309 g) weighing significantly more than the NBO_2_ group (median weight 281 g) (*p =* 0.0001).

**Table 1:** Baseline characteristics and outcomes

	Control (*n* = 18)	NBO_2_ (*n* = 18)	HBO_2_ (*n* = 19)	*P*-value
Weight, gram (SD)	296 (24)	281 (14)	309 (25)	< 0.0001
Temp. at beginning of OP, C° (SD)	36.2 (0.7)	36.4 (0.9)	36.2 (0.6)	0.54
Temp. at end of operation, C° (SD)	36.7 (0.6)	36.4 (0.4)	35.4 (0.4)	0.97
Operation time, minutes (IQR)	50 (45–58)	47 (34–56)	50 (40–64)	0.37
Caecum ligated, millimeters (SD)	62.9 (6.7)	60.8 (6.5)	60.3 (6.8)	0.81
Microorganism^a^				
Positive blood culture	4 (57)	4 (44)	9 (82)	0.16
Polymicrobial	2 (29)	3 (33)	3 (27)	0.77
*Escherichia coli*	4 (57)	3 (33)	6 (55)	0.41
Enterococcus faecalis	1 (14)	3 (33)	2 (18)	0.62
Other	2 (29)	3 (33)	4 (36)	0.89
Cytokines^b^, pg/mL				
TNF-α	57.2 (0.4–114.9)	11.9 (0.4–118.7)	25.7 (0.4–102.9)	0.99
IL-6	33.4 (2.8–70.0)	2.8 (2.8–153.2)	2.8 (2.8–30.5)	0.87
IL-10	79.4 (0.1–155.9)	0.1 (0.1–278.8)	64.7 (0.1–148.5)	0.66
Blood drawn sublingually^c^				
No of subjects	7 (17)	10(22)	9(17)	
Mortality				
24-h	6 (33)	8 (44)	4 (21)	0.13
48-h	10 (56)	9 (50)	8 (42)	0.63
72-h	11 (61)	9 (50)	8 (42)	0.63

*CLP* cecal ligation and puncture, *NBO*_*2*_ normobaric oxygen, *HBO*_*2*_ hyperbaric oxygen, *Temp*. temperature, *IL* interleukin, *TNF* tumor necrosis factor, *SD* standard deviation, *IQR* interquartile range, *OP* operation

Differences between the NBO and HBO groups were tested using Chi-square test/Fisher’s exact test, student’s unpaired t-test or Mann-Whitney U-test

^a^Blood sampling on rats alive at time = 72 h (control *n* = 7; NBO *n* = 9; HBO *n* = 11). Values are reported as number of subjects (%)

^b^Baseline cytokine concentrations taken after CLP procedure at time = 0 h. Values are reported as mean (IQR)

^c^No of samples drawn sublingually. In total 26 of 117 cases, the femoral vein catheter was obstructed. Values are reported as number of subjects (%)

### Mortality

In total, there was a 42% mortality (8 died out of 19) during the observation period in rats treated with HBO_2_, whereas we saw 50% mortality (9 died out of 18) in rats treated with NBO_2_ group and 61% mortality in the control group (11 died out of 18). Treatment with 2.5 bar p_abs_ HBO_2_ did not alter the survival significantly compared with NBO_2_ treatment at any hours (24 h, *p* = 0.13, 48 h, *p* = 0.63 and 72 h, *p* = 0.63). Additionally, we found no statistically significant difference in cumulative survival between the HBO_2_ group and the NBO_2_ group (*p* = 0.59, log-rank test), between the HBO_2_ group and the control group (*p* = 0.24, log-rank test) or between the NBO_2_ group and the control group (*p* = 0.59, log-rank test) (Fig. 2).

### Blood cultures

The predominant bacteria isolated included *Escherichia coli* (*E. coli*) and Enterococcus Faecalis (E. faecalis) (Table 1). Other bacteria isolated included Enterococcus Gallinarum, *Enterobacter cloacae* (*E. cloacae*), Proteus Mirabilis, Staphylococcus Sciuri, Ochrobactrum Anthropi and Streptoccocus Suis. The results showed no statistically significant difference in the number of surviving rats with a positive blood culture at hour 72 in the NBO_2_ group (44%, 4/9) compared with the HBO_2_ group (82%, 9/11) (*p* = 0.16) and no statistically significant difference between the groups according to the number of animals presenting with polymicrobial blood cultures (*p* = 0.77) (Table 1).

### Cytokines

Tables 2, 3 and 4 show the differences in the median cytokine concentration at hour 0, 24, 48 and 72. The difference between HBO_2_ and both NBO_2_ and control groups was calculated for all three cytokines. We found a statistically significant difference between HBO_2_ (489.4 pg/mL; IQR 332.7–650.1) and control /228.6 pg/mL; IQR 40.4–313.2) according to the anti-inflammatory IL-10 at hour 48, however this was not the case for the other time points (hour 0, *p* = 0.82; hour 24, *p* = 0.94; hour 48, *p* = 0.01; hour 72, *p* = 0.60). We found no statistically significant difference between HBO_2_ and control at any time point according to the pro-inflammatory IL-6 (hour 0, *p* = 0.06; hour 24, *p* = 0.85; hour 48, *p* = 0.97; hour 72, *p* = 0.86) and TNF-α (hour 0, *p* = 0.48; hour 24, *p* = 0.85; hour 48, *p* = 0.36; hour 72, *p* = 0.72). Neither did we find any statistically significant difference between HBO_2_ and NBO_2_ at any time point according to the pro-inflammatory IL-6 (hour 0, *p* = 0.87; hour 24, *p* = 0.98; hour 48, *p* = 0.91; hour 72, *p* = 0.94) and TNF-α (hour 0, *p* = 0.99; hour 24, *p* = 0.88; hour 48, *p* = 0.97; hour 72, *p* = 0.94). This was also the case for the anti-inflammatory IL-10 (hour 0, *p* = 0.66; hour 24, *p* = 0.31; hour 48, *p* = 0.13; hour 72, *p* = 0.37).

**Table 2:** Differences in median TNF-α concentration

Hours	HBO_2_	NBO_2_	Control
0	25.7 (0.4–102.9)	11.9 (0.4–188.7)	57.2 (0.4–114.9)
24	59.4 (0.4–138.5)	33.5 (0.4–136.3)	64.0 (0.4–157.8)
48	9.5 (0.4–74.0)	0.4 (0.4–93.2)	47.0 (1.2–137.6)
72	55.8 (0.4–122.4)	62.1 (0.4–125.5)	5.1 (0.4–131.8)

*TNF*-α tumor necrosis factor alfa, *CLP* cecal ligation and puncture, *NBO*_*2*_ normobaric oxygen treatment, *HBO*_*2*_ hyperbaric oxygen treatment

Values denote medians with IQR. Comparisons were performed using the Mann-Whitney U-test. No significant differences (*p* < 0.05) were found comparing HBO_2_ with NBO_2_ and HBO_2_ with control

**Table 3:** Differences in median IL-6 concentration

Hours	HBO_2_	NBO_2_	Control
0	2.8 (2.8–30.5)	2.8 (2.8–153.2)	33.4 (2.8–70.0)
24	161.5 (14.6–296.5)	197.9 (2.8–278.6)	144.3 (53.4–322.9)
48	2.8 (2.8–77.9)	2.8 (2.8–101.3)	2.8 (2.8–95.5)
72	5.5 (2.8–134.9)	2.8 (2.8–224.7)	2.8 (2.8–182.6)

*IL* interleukin, *CLP* cecal ligation and puncture, *NBO*_*2*_ normobaric oxygen treatment, *HBO*_*2*_ hyperbaric oxygen treatment

Values denote medians with IQR. Comparisons were performed using the Mann-Whitney U-test. No significant differences (*p* < 0.05) were found comparing HBO_2_ with NBO_2_ and HBO_2_ with control

**Table 4:** Differences in median IL-10 concentration

Hours	HBO_2_	NBO_2_	Control
0	64.7 (0.1–148.5)	0.1 (0.1–278.8)	79.4 (0.1–155.9)
24	591.0 (336.2–969.1)	405.8 (296.6–579.9)	517.2 (289.6–1138)
48	489.4 (332.7–650.1)*	337.8 (203.6–453.4)	228.6 (40.4–313.2)*
72	250.9 (99.4–330.5)	72.5 (0.1–538.5)	169.5 (585.8–0.1)

*IL* interleukin, *CLP* cecal ligation and puncture, *NBO*_*2*_ normobaric oxygen treatment, *HBO*_*2*_ hyperbaric oxygen treatment

Values denote medians with IQR. Comparisons were performed using the Mann-Whitney U-test

*: *p* = 0.01 comparing parameter in HBO_2_ vs. control. No other significant differences (p < 0.05) were found comparing HBO_2_ with NBO_2_ and HBO_2_ with control

We assessed the concentration of cytokines on hour 24 on all animals alive longer than 24 h after CLP. We grouped them in survivors (alive until 72 h after CLP) and non-survivors (died between 24 and 72 h after CLP). We found non-survivors to have a significant higher IL-6 concentration at 24 h compared to survivors (non-survivors IL-6302.3 pg/mL (102.8–581.6) vs. survivors IL-6 = 107.9 pg/mL (20.5–252.2), (*p* = 0.04)). There was no statistically difference in the concentration of TNF-α and IL-10 between survivors and non-survivors (non-survivors TNF-α = 59.37 pg/mL (0.37–179.8) vs. survivors TNF-α 37.52 pg/mL (0.37–122.6), *p* = 0.31; non-survivors IL-10 = 1050 pg/mL (274.1–5318) vs. survivors IL-10 = 450.2 pg/mL (342.2–627.4), *p* = 0.13).

### Discussion

In this study, we evaluated delayed intervention of hyperbaric oxygen as a treatment for sepsis. We found no difference in the concentrations of IL-6, TNF-α and IL-10 between the groups treated with hyperbaric or normobaric oxygen during the first 72 h of sepsis. However, we found a significantly higher concentration of IL-10 at hour 48 in the HBO_2_ group compared with the control group, but not at any other time points. Moreover, we found a significantly higher concentration of IL-6 at hour 24 in non-survivors compared to survivors. Lastly, we found no difference in survival rates between all the treatment groups.

In previous reports, the experimental animals were either terminated or anesthetized at varied time points in order to collect samples and to obtain knowledge of cytokine concentrations at different time points [8, 19, 25, 26]. This weakens longitudinal conclusions and results in a larger amount of animal sacrifice. In the present study, each rat was monitored with intermittent blood sampling which allows evaluation of the changes in cytokine concentrations on a daily basis and to follow each rat individually. In addition, we were able to reduce the number of rats by 75% and still obtain the same number of samples, which gives this study an enormous ethical advantage. Moreover, it is a strength that we have combined the gold standard of sepsis induction models with intermittent blood sampling in the individual rat making the model applicable to clinical practice with respect to the study of different interventions, alone or in combination [20].

The present study uses only clinical signs and blood cultures to ensure that the animals are septic, since earlier studies have shown that CLP imitates sepsis according to vascular derangement, alterations in the metabolic state and with clinical signs showing disease [15, 20, 27]. In this study we found that 63% of the blood cultures were positive (Table 1) with *E. coli*, E. Faecalis and *E. cloacae* as the most frequent bacteria isolated, corresponding to peritonitis from fecal matter. A post mortem laparotomy was performed in all animals, disclosing a necrotic cecum tightly adherent to close-lying anatomical structures, including signs of peritonitis with excessive cloudy intraperitoneal fluid combined with a foul smell. The information from the clinical symptoms and signs in combination with the positive blood cultures and the laparotomy findings suggest that all rats were septic.

Sepsis is a critical illness currently treated with fluids and antibiotics. Turnbull et al. has demonstrated that antibiotics can improve the outcome in murine sepsis lowering overall mortality, but if a concentration of 14,000 pg/mL of IL-6 is reached, the animals are destined to die despite antibiotic treatment [28]. High plasma concentrations of IL-6 in humans are also associated with the development of septic shock and is a predictor of mortality [5, 29–31]. The current experiment confirms that IL-6 was a predictor of death with a significantly higher concentration of IL-6 in non-surviving animals at 24 h indicating a state of septic shock (Table 5). As shown by Singleton KD and Wischmeyer PE, CLP distance and size of needle puncture determines mortality. Performing a 30% CLP with a 16G double-puncture as in the current experiment should give a 90% mortality after 72 to 96 h [19]. Accordingly, the severity of the model presented here, in combination with the high expression of IL-6 at 24 h measured before oxygen intervention, indicates this study applies to a severe state of sepsis.

**Table 5:** Difference between survivors and non-survivors in median cytokine levels at hour 24

	Non-survivors	Survivors	*P*-value
TNF-α	59,37 (0.37–179.8)	37.52 (0.37–122.6)	0.31
IL-10	1050 (274.1–5318)	450.2 (342.2–627.4)	0.13
IL-6	302.3 (102.8–581.6)	107.9 (20.5–252.2)	0.04*

Survivors defined as rats alive until 72 h after CLP, non-survivors defined as rats dead between 24 and 72 h after CLP

*IL* interleukin, *TNF*-α Tumor necrosis factor alfa, *CLP* cecal ligation and puncture

Values denote medians with IQR. Comparisons were performed using the Mann-Whitney U-test

*p= 0.04 comparing parameter in survivors vs. non-survivors. No other significant differences (*p* < 0.05) were found

It has been suggested that HBO_2_ might exert its effect through production of the anti-inflammatory IL-10, which acts by lowering the pro-inflammatory IL-6 and thereby reducing mortality [5, 7, 18, 29, 30]. IL-10 has furthermore been demonstrated to play a critical step in the progression to a lethal state of sepsis and that endogenous production of IL-10 delays the onset of mortality in CLP induced sepsis [18]. In the present study, we found a significantly higher concentration of IL-10 at hour 48 in the HBO_2_ group compared with control. This finding is in accordance to previous studies showing that HBO_2_ treatment might boost the production of IL-10 [7]. In this study, we found no statistical difference in the mortality between the HBO_2_ group and the control group, hence the statistically higher concentration of IL-10 in the HBO_2_ group at 48 h did not influence survival. Interestingly, we found no significant difference between HBO_2_ and NBO_2_ in the concentrations of IL-6, TNF-α and IL-10 at any hours. Overall, the concentration of all three measured cytokines decreased after the first 24 h. This is probably due to the most severely ill animals dying within the first 24 h of the study. The cytokine value on hour 24 and forth may therefore represent less ill animals, explaining the lower values of both pro- and anti-inflammatory cytokines as measured. As later described, the cytokine concentrations on Day 0 fluctuated greatly and may have contributed to the lack of statistically significant results in the measured cytokines.

Buras et al. examined the effect of HBO_2_ treatment according to survival in septic mice and found that two daily treatments at 2.5 atm with a 12-h interval and a total of eight treatments were required to reduce mortality [7]. They also found that NBO_2_ had no effect in terms of survival. In the present study, HBO_2_ was delayed by 24 h after CLP-induction of sepsis, whereas Buras et al. administered two HBO_2_ sessions within the first 24 h [7]. Undoubtedly, the 24 h delay of the HBO_2_ treatment were fatal to overall survival in this CLP model, although the treatment pressure was chosen with reference in Buras et al.’s studies [7]. The delay of treatment was chosen to study the rats in a circulatory hypodynamic state of sepsis, which follows the initial hyperdynamic state and subsequently progresses into a state of severe sepsis [32]. Experimentally, early intervention with hyperbaric oxygen has been shown to ameliorate sepsis. In most studies, however, the treatment is given shortly after induction of sepsis before the signs of manifest sepsis appear [7, 14]. In clinical practice, however, we cannot treat sepsis before the symptoms and signs appear. Moreover, logistic challenges and patient transfer between hospitals and other procedures can delay hyperbaric oxygen treatment. Therefore, the chosen delay of the hyperbaric treatment in this report reflects clinical practice. However, the delay also constitute a limitation since many animals die before the first intervention.

There are some other limitations of the present study. Firstly, no sample size calculation was performed prior to the experiment, which increases the risk of a type II error due to a potential insufficient number of animals included in each group. However, others studies have investigated sepsis and outcomes in animals using similar sample size, which is why we chose the number of animals in the current study [7, 15, 18]. Secondly, the cytokine concentrations on Day 0 varied greatly (see Tables 2, 3 and 4 for concentrations). It is well known in animal models to see a stress response from anesthesia, which can vary between the animals, however in the current study it varied greater than we expected. It is important to outline, that the baseline sample was taken prior to CLP hence no infection was present yet. The fluctuating concentrations on Day 0 may have contributed to the lack of statistically significant results in cytokines concentrations between groups. Thirdly, although rats were randomized by groups a difference occurred in rat weight at the time of inclusion (Table 1). Minor changes in weight (< 10%) induced by high fat diet has earlier been shown to be associated with increased oxidative stress and inflammation causing increased rat mortality in CLP models of sepsis [33, 34]. However, our rats were all fed standard chow so a worsening effect of the weight is not to be expected. The weight differences could be related to differences in age at the time of randomization, but all rats were provided similar acclimatization of 1½ week and previous reports have failed to find any correlation between age, weight and final outcome in similar CLP models [35]. Finally, as the Kaplan-Mayer curves show (Fig. 2) most of the mortality occurred during the first 24 h and therefore before the various treatment modalities were commenced. This means that NBO_2_ and HBO_2_ therapies were studied in a small number of rats, hence the size of the surviving group may have been too small to demonstrate a possible beneficial effect. Measurements of other biomarkers e.g. nuclear protein high mobility group box protein 1 (HMGB1) has emerged in sepsis, and we cannot exclude other biomarkers to link to survival in our model [36].

## Conclusion

This study documents the endogenous production of IL-6, TNF-α and IL-10 in rats with cecal ligation and puncture (CLP) induced sepsis on conscious animals with intermittent blood sampling in the individual rat during a 72-h period. We found a significant higher level of IL-6 in non-surviving animals, suggesting IL-6 to be a potential biomarker predicting death in sepsis. Moreover, we found a statistically higher concentration of the IL-10 in the HBO_2_ group at hour 48 versus control animals, but the elevation of the anti-inflammatory cytokine did not have a statistically significant effect on mortality. This might be due to the 24 h delay of the HBO_2_ treatment. We found no additional statistically significant differences in the cytokine response between HBO_2_, NBO_2_ and control. Additionally, no statistically significant differences were seen in mortality for any group comparisons. Delayed and infrequent intervention in the late phase of septic shock may compromise the previously reported ameliorating effects of HBO_2_ treatment. The model presented in this study provides a template for further preclinical studies in the field of hyperbaric oxygen treatment for sepsis and future studies should consider earlier intervention with respect to hyperbaric oxygen effects.

## Abbreviations

CLP: Cecal ligation and puncture
*E. cloacae*: *Enterobacter cloacae*
*E. coli*: Escherichia Coli
E. faecalis: Enterococcus Faecalis
HBO_2_: Hyperbaric oxygen treatment
HMGB1: High mobility group box protein 1
IL: Interleukin
NBO_2_: Normobaric oxygen
ROS: Reactive oxygen species
TNF-α: Tumor necrosis factor alfa
